# Pollinators on Cowpea *Vigna unguiculata*: Implications for Intercropping to Enhance Biodiversity

**DOI:** 10.3390/insects12010054

**Published:** 2021-01-11

**Authors:** Beatrice N. Dingha, Louis E. Jackai, Barbara A. Amoah, Clement Akotsen-Mensah

**Affiliations:** 1 Department of Natural Resources and Environmental Design, North Carolina A&T State University, Greensboro, NC 27411, USA; lejackai@ncat.edu (L.E.J.); baamoah@ncat.edu (B.A.A.); 2 Cooperative Extension, Lincoln University, Jefferson City, MO 65101, USA; akotsen-mensahc@lincolnu.edu

**Keywords:** cowpea, *Vigna unguiculata*, pollinator decline, intercropping, flower resources, pollinator

## Abstract

**Simple Summary:**

Pollinators are a major part of global biodiversity and they provide ecosystem services important for the production of many crops. Their abundance and diversity have declined steadily in recent years. Loss of foraging resources through degradation and fragmentation of natural habitats has been a major factor. Enhancing floral resources in the environment can mitigate this decline. Cowpea nectar has been reported to make the crop attractive to pollinators. We evaluated twenty-four cowpea varieties for pollinator abundance and diversity using pan traps, sticky traps, and direct visual counts. Sticky traps captured the highest number of pollinators and pan traps the least. The highest number of pollinators was recorded on Penny Rile cowpea and the lowest on Iron and Clay which had no flowers. Whippoorwill had the most flowers and ranked third in number of pollinators. Our findings indicate that cowpeas can be used to improve pollinator efficiency. Intercropping pollinator-dependent crops with cowpea varieties such as Penny Rile, Dixielee, and Whippoorwill will not only provide resources for the pollinators but can also be effective in increasing pollinator number and activity to increase crop yields.

**Abstract:**

Pollinators are on the decline and loss of flower resources play a major role. This raises concerns regarding production of insect-pollinated crops and therefore food security. There is urgency to mitigate the decline through creation of farming systems that encourage flower-rich habitats. Cowpea is a crop that produces pollen and nectar attractive to pollinators. Twenty-four cowpea varieties were planted, and the number of pollinators were counted using three sampling methods: pan traps, sticky traps, and direct visual counts. Five pollinator types (honey bees, bumble bees, carpenter bees, wasps, and butterflies and moths), 11 and 16 pollinator families were recorded from direct visual counts, pan and sticky traps, respectively. Pollinator distribution varied significantly among varieties and sampling methods, with highest number on Penny Rile (546.0 ± 38.6) and lowest (214.8 ± 29.2) in Iron and Clay. Sticky traps accounted for 45%, direct visual counts (31%), and pan traps (23%) of pollinators. Pollinators captured by pan traps were more diverse than the other methods. The relationship between number of pollinators and number of flowers was significant (*r*^2^ = 0.3; *p* = 0.009). Cowpea can increase resources for pollinators and could be used to improve pollinator abundance and diversity in different farming systems.

## 1. Introduction

Pollinators are a key component of global biodiversity, providing vital ecosystem services to crops and wild plants. There is evidence that pollination of crops benefit food production through yield quantity and/or quality [1,2,3,4]. Globally, pollinators are responsible for pollinating approximately 30,000 plant species [5]. Among these plant species, 75% are crops that benefit directly or indirectly from the ecosystem service provided by pollinators [1]. In recent years, substantial declines in the abundance and diversity of insect pollinators have been widely documented [6,7,8,9]. Of concern is the honey bee, which pollinates approximately USD 10 billion worth of crops in the USA annually [10]. A number of factors including the degradation and fragmentation of natural habitats [11], the loss of flower-rich plant communities associated with traditional landscape uses such as heathlands and legume-based set aside fields [12], the spread of pathogens and parasites [13], and the inappropriate and widespread use of agricultural pesticides [14], have contributed to this decline. Among these factors, habitat loss, degradation and fragmentation of natural habitat through agricultural intensification have been recognized as the main causes of decline in local and global biodiversity [15]. Unfortunately, this also affects native and wild bees that have been documented to play an integral ecological role as pollinators of a large number of wild flowers and cultivated crops [16,17,18].

As a result of these findings, the United States Department of Agriculture [19] has promoted the creation of flower-rich habitats in the form of hedgerows, field-border plantings, temporary flowering cover crops and flower-rich buffer strips. Despite the positive effects of adjacent natural habitats resulting in increased pollinator abundance the application has been with non-edible crops (such as ornamental shrubs and trees) with no demonstrated study on additional profit to growers, which has hindered the adoption of this strategy. Several vegetable crop species including cowpea, *Vigna unguiculata* (L.) Walp., have been reported to be attractive to pollinators [19]. Cowpea, also known as blackeye pea, is an important vegetable crop grown widely by small-medium scale growers and also on large commercial scale in the southern USA. The crop is utilized both as a vegetable crop and a dry bean. It is adapted to a wide range of growing conditions and considered one of the best drought-resistant food crops [20,21] and, therefore, it can help counter the negative impact of climate change, which can undermine agricultural production and sustainability [22,23]. It is important for the sustainability of soil fertility [24,25] because of its ability to fix atmospheric nitrogen. In addition, cowpea exhibits different morphological attributes including floral resources that enhances honey bees and other bee species attraction to it [26]. An increasing body of empirical evidence shows that the management of floral resources in local agricultural fields can mitigate adverse human impact on pollinator diversity and pollination services [27,28,29,30]. However, cowpea has traditionally been grown as an intercrop with reported increase in productivity; for example, tomato with cowpea [31], amaranth with cowpea, cucumber with cowpea [32], maize with cowpea [33], and cassava with cowpea [34,35]. These studies indicated that intercropping or companion cropping produced more grain, tuber, and biomass yield than sole cropping. Increased yield was attributed to benefits derived from intercropping (improve soil fertility, control of some pests and favorable environment for beneficial insects) and not to those associated with floral resources of cowpea.

Cowpea floral resources, especially nectar, are attractive to pollinators [26]. Information on the successful use of cowpea as an intercrop for the purpose of enhancing pollinator activity and increase crop production is lacking. In recognition of these gaps, we conducted field experiments with the main objective to identify cowpea varieties highly attractive to pollinators and the pollinator diversity associated with each variety. We used three sampling methods: sticky traps, pan traps, and direct visual counts, which have been compared and reported in other studies to vary in their effectiveness depending on the pollinator type [36,37,38,39]. Therefore, to have a better knowledge of the diversity and abundance of pollinators associated with the cowpea varieties, all three sampling methods were used.

## 2. Materials and Methods

### 2.1. Study Site and Experimental Designs

In 2017, a field experiment was carried out at the North Carolina Agricultural and Technical State University (NCA & TSU) Research Farm in Greensboro, North Carolina, USA (36.0586243° N, 79.7358932° W). The treatments used for this study included twenty-four cowpea varieties (Big Boy, Big Red Ripper, Black Crowder, Carrapichio, California Blackeye5, Cream 40, CT Pinkeye Purple Hull, Dixielee, Early Scarlet, Iron and Clay, Lady, Mayo Colima, Mississippi Silver, Peking Black, Penny Rile, Purple Hull, Big Boy, Red Bisbee, Rouge et noir, Running Conch, Tohono O’odham, Vietnamese Black, Whippoorwill, Whippoorwill Steel Black, and Zipper Cream). These cowpeas are among the most popular varieties in many southern states. Seeds were obtained from commercial seed houses. The experiment was set up as a randomized complete block design (RCBD) with four replications and each experimental unit consisted of two 5 m row of each cowpea variety. The seeds were planted manually at 0.01–0.015 m below the soil surface, 0.4 m apart in row and 1 m between rows from 22 to 25 May 2017. General agronomic practices such as weed control (weed eater) and irrigation (drip irrigation system) were carried out as needed. No insecticide and fertilizer were applied.

### 2.2. Sample Collection

At the beginning of flowering, plants were monitored daily. For each cowpea variety, data was recorded on the number of flowers and the number of days to first flower. The number of days to when the first pod was observed was also recorded for each variety.

Three sampling methods (pan traps, sticky traps, and direct visual counts) were used to sample pollinators. Cowpeas flowering started six weeks after planting. Sampling took place on 3, 10 and 14 July 2017 for sticky traps, pan traps, and direct visual counts, respectively, and continued weekly for five weeks.

#### 2.2.1. Number of Flowers and Yield Data

A total of six weeks after planting, five random flowering plants for each variety were selected and the number of flowers on each plant counted and recorded. A total of twenty-five fresh pods at the R6 stage (50% of pods with fully developed seeds) from each cowpea variety were harvested and weight recorded.

#### 2.2.2. Evaluation of Pollinators Using Pan Traps

Pollinators were sampled using three colored (blue, white, and yellow) pan traps. Sampling began on 10 July 2017 and was repeated weekly for five weeks. Traps consisted of 16 oz. squat polypropylene deli bowls (BioServ, Frenchtown, New Jersey, United States of America) painted with UV-bright fluorescent blue (blue trap) or yellow paint (yellow trap) and unpainted 12 oz. white styrofoam bowls (white trap) (Uline, Pleasant Prairie, WI, USA). Trap setup was made by gluing individual unpainted 16 oz. polypropylene deli bowls onto a 36” plant prop and three of these placed 0.2 m apart between the 2 rows of each treatment during the entire sampling period. Each colored bowl (blue, yellow, and white) was then placed inside one of the unpainted bowls on the prop and filled with approximately 250 mL of soapy water solution (2.5 mL of detergent in 1-gallon water). The traps were placed such that they were at the same level as the crop canopy. Traps were set out weekly from 13 July to 10 August between 8:00–10:00 a.m. and removed after 24 h. Each colored pan trap was drained, and contents placed in vials containing 70% ethanol and taken to the laboratory where they were stored in a refrigerator for later identification with a microscope (AmScope Stereozoom trinocular microscope, SZMT2 Series, WF10X/20; United Scope LLC, Irvine, CA, USA) to the family level. Traps were collected in the order they were placed to ensure that all traps were available to insects for roughly the same duration.

#### 2.2.3. Evaluation of Pollinators Using Sticky Traps

Sticky trap sampling began on 3 July 2017 and was carried out by placing one two-sided yellow sticky trap (8 × 13 cm^2^) (PestrapTM, Phytotronics Inc., Rider Trail North Earth City, MO, USA) adhered to a metal plant stake and placed at the center of each 5 m row. This was aimed at capturing smaller insects that may have been missed with visual and pan trap sampling. They also provided an alternative sampling method for comparison. We adjusted trap height to keep them just below canopy height. Traps were removed after 24 h and replaced weekly from 3 July to 9 August. All samples were transferred into labelled Ziploc^®^ bags and taken to the laboratory where they were identified to the family level using a microscope (AmScope Stereozoom trinocular microscope, SZMT2 Series, WF10X/20; United Scope LLC, Irvine, CA, USA).

#### 2.2.4. Evaluation of Pollinators from Direct Visual Counts

Direct visual counts began on 14 July 2017. The number of pollinator visitors on the two 5 m rows of each cowpea variety was identified and quantified using “snapshot” counts, in which the number of insects were counted for 60 s through direct visual counts. Pollinator types that were observed and recorded were bumble bees, carpenter bees, honey bees, butterflies and moths, and wasps. Weekly observational snapshots were conducted for pollinators between 9:00 and 11:00 a.m. for five weeks from 4 July to 8 August. As insects generally stayed on the same variety for a few minutes during their foraging behavior, this implies that the same insect was unlikely to have been counted twice.

### 2.3. Data Analysis

All analyses were conducted using JMP Pro (JMP Pro v. 14 SAS Institute, Cary, NC, USA). For each sampling method, cowpea variety, weekly totals were calculated for pollinator types/families and used to determine abundance per treatment. The data were first tested for the assumptions of ANOVA. Where, the assumptions of ANOVA were violated, the data were further transformed and tested again for the assumptions of ANOVA. Since the transformed data improved the data in terms of meeting the assumption of normal distribution, the transformed data were then analyzed using a mixed model with sampling week, sampling method (pan trap, sticky trap and visual), treatment (cowpea variety), and the interactions between sampling method and treatment as fixed effects and sampling week as a random effect. If the interaction between sampling week, sampling method and treatment was not significant, the data were pooled (seasonal total) and analyzed by sampling week, sampling method and treatments using ANOVA. On the other hand, if interaction between sampling week, sampling method, and treatment was significant, the data was analyzed separately for each sampling week for the trap type and treatment using ANOVA. Means were separated using Tukey–Kramer Honestly Significant Difference at *p* = 0.05. The relationships between the number of flowers and the number of pollinators and between the number of pollinators and fresh pod weight were described using regression analysis.

The diversity of pollinators reported from pan traps, sticky traps, and through direct visual counts of different cowpea varieties was assessed using the Shannon–Weaver Diversity Index (H′) [40]. The Index is expressed as: H′ = −∑ *pi* ln(*pi*)

where H’ = Shannon–Weaver diversity index

*pi* = the proportion of individuals found in species *i*

*pi* estimated as: *pi* = ni/N

where ni = number of individuals in species i

N = total number of individuals in the community

The Shannon Equitability (Evenness) Index (E) normalizes the Shannon Diversity Index (H’) to a value between 0 and 1. It provides an idea about the evenness of the distribution of groups of organisms in a community. An index value of 1 means that all groups have the same frequency.

The Evenness Index (E), is expressed as: E = H′/log(k)

where E = Evenness,

H′ = Shannon–Weaver Diversity Index

k = number of species/groups in the community

For each sampling method (direct visual counts, pan trap and sticky trap), the total number of pollinators in each family or pollinator types captured on each variety for the entire sampling period was used to calculate the Diversity and Evenness indices.

## 3. Results

### 3.1. Plant Parameters

The number of days to flower, number of flowers, days to first podding, and color of flowers for each variety are presented in Table 1. The number of days to flower was significantly different (*F*_21, 66_ = 2.83, *p* = 0.0007) among the varieties. The first flower was observed on Early Scarlet 42.0 ± 1.2 days after planting. Purple Hull Big Boy and CBE5 were the last to flower at 55.5 ± 2.1 and 55.5 ± 3.7 days after planting, respectively. There were significant differences (*F*_23, 456_ = 22.05, *p* < 0.0001) among the varieties in the number of flowers recorded. No flower was recorded on Tohono O’Odham and Iron and Clay varieties. The lowest number of flowers were recorded on CBE5 (18.6 ± 1.5) and Purple Hull Big Boy (19.2 ± 1.3). Whippoorwill (56.4 ± 4.2) and Early Scarlet (55.5 ± 3.9) had the highest number of flowers. It was observed that most of the varieties had purple flowers with about a third producing white flowers (Table 1). Early Scarlet was the only variety that produced yellow flowers. The number of days to first pod which ranged from 57.5 ± 2.5 days in Early Scarlet and Ct Pinkeye Purple Hull, to 64.5 ± 2.5 in Carrapichio, CBE5, Cream 40, Purple Hull Big Boy, and Red Bisbee, was not significantly different among varieties (*F*_21, 66_ = 0.84, *p* = 0.66).

### 3.2. Effect of Sampling Week, Sampling Method and Cowpea Variety on Pollinator Abundance

The mixed model analyses revealed significant effects of week, sampling methods and treatment effect on pollinators captured (Table 2). Based on the recorded significant treatment (abundance) effects, pollinator abundance was separately compared for sampling methods and treatment. The ANOVA revealed a significant effect of pan trap and direct visual counts treatment on pollinator abundance.

### 3.3. Abundance and Diversity of Pollinators Associated with Cowpea Varieties

#### 3.3.1. Evaluation of Pollinators Using Pan Traps

Eleven pollinator families were identified from the three colored pan traps deployed on the twenty-four cowpea varieties. These were Andrenidae, Apidae, Crabronidae, Formicidae, Halictidae, Hesperiidae, Pompilidae, Sphecidae, Syrphidae, Tachinidae, and Vespidae. Of these families, Apidae, Crabronidae, Halictidae, and Tachinidae, were the four most abundant families (Table 3). The other seven pollinator families were the least abundant (≤0.03) on each cowpea variety and were not included in Table 3. Halictidae was the most abundant family in each colored pan trap and cowpea variety. The mean number of Halictids caught in white pan trap among the cowpeas ranged from 20.0 ± 2.9 on Zipper Cream to 39.8 ± 9.6 on Whippoorwill Steel Black; this was not significantly different among varieties. Hesperiidae was the least (0.3 ± 0.3) abundant family and was only identified from white traps placed on California Black Eye-5 (CBE-5) and on Penny Rile. From the white pan trap, Apidae was the only pollinator family that had significant difference (*p* = 0.02) among the 24 cowpea varieties with high numbers 10.0 ± 4.1, 8.5 ± 3.3 and 6.5 ± 4.6 recorded on Penny Rile, Whippoorwill Steel Black and Dixielee, respectively. Even though Halictidae was the most abundant pollinator family in the yellow pan trap on each of the 24 cowpea varieties, this number was significantly different (*p* = 0.02) among the cowpea varieties with more recorded on Penny Rile (17.5 ± 49) and Black Crowder (17.3 ± 3.2). In addition, there was a significant difference (*p* = 0.03) in the number of Crabronidae among the varieties with the highest capture recorded on Penny Rile (7.3 ± 2.1) and Dixielee (5.5 ± 1.8). For all the insect families captured in the blue colored pan trap, there was no significant difference in the number of pollinators recorded among the 24 cowpea varieties.

**Figure 1:**
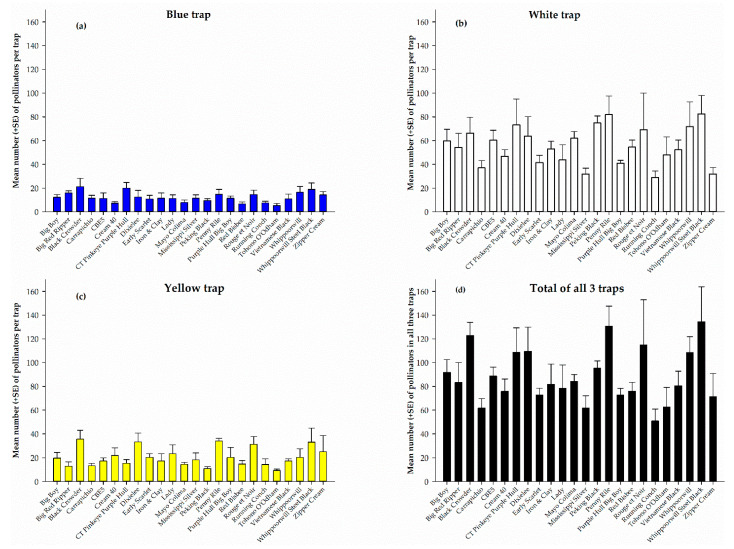
Pollinators captured in blue (**a**), white (**b**), and yellow (**c**) pan traps and total pollinators in all three traps (**d**) on different cowpea varieties for the entire sampling period.

The total pollinators captured from each cowpea variety in the white, yellow, and blue pan traps and pollinators from all three traps combined during the five-week sampling period is shown in Figure 1. White pan trap captured the highest number of pollinators on each of the 24 cowpea varieties (Figure 1b) compared to the blue- and yellow-colored traps. However, the catch was not significantly different (*F*_23, 72_ = 1.52, *p* = 0.09) among the varieties nonetheless, Whippoorwill Steel Black recorded the highest number (82.5 ± 15.6) and Running Conch the lowest (29.0 ± 5.4). Among the traps, blue pan trap recorded the lowest number of pollinators on each of the 24 cowpeas (Figure 1a) with no significant difference (*F*_23, 72_ = 1.31, *p* = 0.19) in the number of pollinators among the varieties. The numbers ranged from 5.3 ± 1.7 on Tohono O’Odham to 21.3 ± 7.0 on Black Crowder (Figure 1b). Similarly, the number of pollinators recorded in the yellow pan trap was not significantly different (*F*_23, 72_ = 1.63, *p* = 0.06) among the 24 cowpea varieties. However, Black Crowder recorded the highest number (35.5 ± 7.5) and Tohono O’Odham the least (9.3 ± 1.3) (Figure 1c). Combined catches from all three pan traps during the entire sampling period, shows that the total number of pollinators recorded among the cowpea varieties was significantly different (*F*_23, 72_ = 2.02, *p* = 0.01) and ranged from 50.8 ± 10.1 on Running Conch to 134.5 ± 29.3 on Whippoorwill Steel Black (Figure 1d).

**Figure 2:**
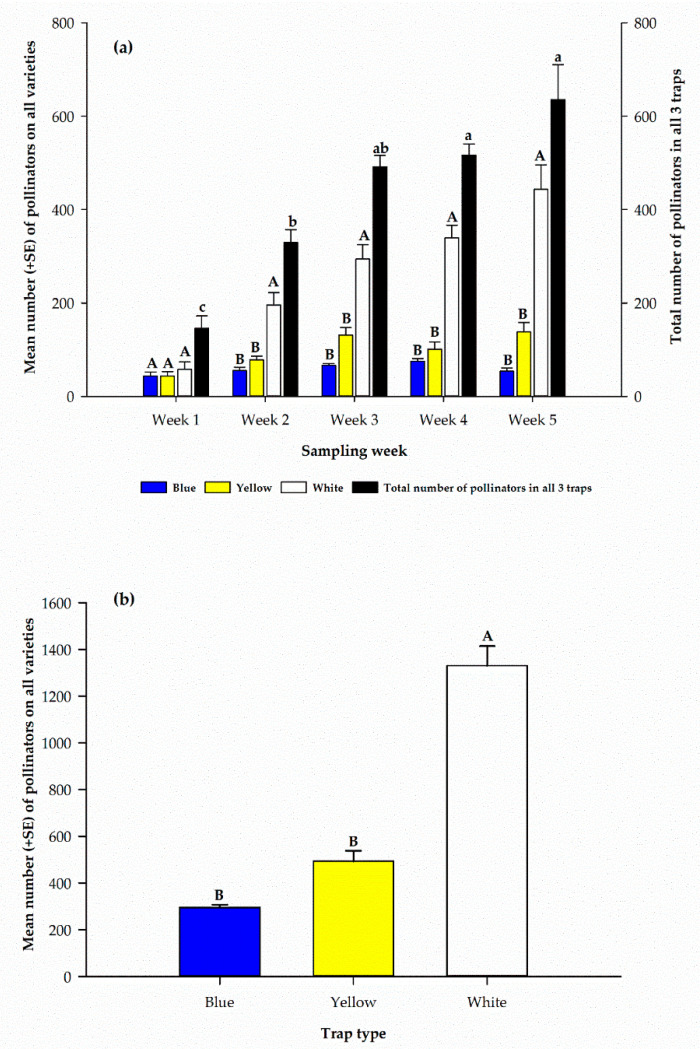
(**a**) Weekly distribution of pollinators in three different color pan traps. (**b**) Total number of pollinators captured in three different traps on cowpea varieties over the five sampling weeks. In (**a**), upper case letters are used to compare different color trap means within each week. Lower case letters are used to compare the combined trap means across all weeks. Mean numbers with the same letters are not significantly different (*p* > 0.05).

Combined pollinator catches for each sampling week indicated a significant difference (*F*_4, 15_ = 21.8, *p* < 0.0001) among the sampling weeks, with a steady increase from the first sampling week (146.0 ± 26.2) to the last (635.3 ± 75.2) (Figure 2a). In the first week, the catch among all pan traps was not significantly different (*F*_2, 9_ = 0.50, *p* = 0.62). However, during the second through the fifth week, white pan traps captured significantly (*p* < 0.01) more pollinators compared to the other traps (Figure 2a). Differences were not significant between blue and yellow traps in the number of pollinators captured during each sampling week. However, within the white pan traps, the least number of pollinators (58.0 ± 15.6) was recorded on the first sampling week and the highest (443.3 ± 52.5) on the fifth. Similarly, among yellow pan traps the least (44.0 ± 9.1) number of pollinators was recorded on the first sampling week and the highest (138.0 ± 20.5) on the fifth (Figure 2a). However, the highest number (75.3 ± 5.9) of pollinators was recorded on the fourth sampling week in the blue pan trap (Figure 2a).

Combined catches for each colored trap during the five sampling weeks indicate there was a significant difference (*F*_2, 9_ = 99.1, *p* < 0.0001), among the three colored pan traps with white pan trap capturing the highest (1330.5 ± 83.6) number of pollinators and the least (295.3 ± 12.3) in blue colored pan trap (Figure 2b).

#### 3.3.2. Evaluation of Pollinators Using Sticky Traps

**Figure 3:**
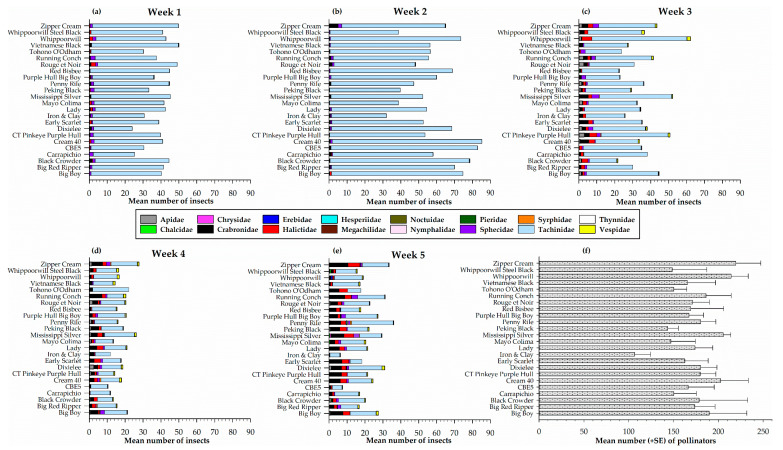
Weekly distribution of pollinator families (**a**–**e**) and total number of pollinators (**f**) on different cowpea varieties in sticky traps.

Pollinators captured on sticky traps on each of the 24 cowpea varieties for each sampling week is presented in Figure 3. A total of sixteen pollinator families were recorded on sticky traps during the sampling period. The families were: Apidae, Chalcidae, Chrysidae, Crabronidae, Erebidae, Halictidae, Hesperiidae, Megachilidae, Noctuidae, Nymphalidae, Pieridae, Sphecidae, Syrphidae, Tachinidae, Tynnidae, and Vespidae. In week one, statistically similar numbers (*F*_23, 72_ = 0.73, *p* = 0.80) of pollinators were recorded among the 24 cowpea varieties, the highest number recorded on Vietnamese Black (50.0 ± 14.1) and Zipper Cream (49.8 ± 6.9) (Figure 3a). This sampling week was the least diverse with seven pollinator families (Crabronidae, Halictidae, Hesperiidae, Pieridae, Sphecidae, Tachinidae, and Vespidae) recorded on the sticky traps (Figure 3a). In the second week, two additional families (Apidae and Erebidae) were recorded for a total of nine pollinator families. During this sampling week, the highest number of pollinators was recorded on Cream 40 (85.3 ± 7.9) a number that was not significantly different (*F*_23, 71_ = 0.89, *p* = 0.61) from what was recorded on the other varieties (Figure 3b). By week three, two new pollinator families (Syrphidae and Thynnidae) were recorded, and the highest number of pollinators recorded on Whippoorwill (62.3 ± 28.2), Mississippi Silver (52.3 ± 9.9), and CT Pinkeye Purple Hull (50.8 ± 16.3) although this was not significantly different (*F*_23, 72_ = 0.91, *p* = 0.59) among the cowpeas at this time (Figure 3c). Similarly, in week four, the highest number of pollinators was recorded on Zipper Cream (27.8 ± 9.1), Mississippi Silver (26.3 ± 7.1), and Tohono O’odham (22.0 ± 8.8) (Figure 3d) that included a new family (Megachilidae). However, there were no significant differences (*F*_23, 72_ = 0.52, *p* = 0.96) among the cowpea varieties. The final sampling week has the most diverse pollinator families recorded (14 total) (Figure 3e). The highest number of pollinators were recorded on Penny Rile (36.0 ± 11.1), Zipper Cream (33.5 ± 5.3), and Running Conch (31.3 ± 5.7) with no significant difference (*F*_23, 72_ = 1.36, *p* = 0.16) among the varieties (Figure 3e).

Overall, the total number of pollinators captured from all 24 cowpea varieties each week was significantly different (*F*_4, 15_ = 11.0, *p* = 0.0002) among the five sampling weeks with the lowest (423.5 ± 67.8) in week four followed by (522.5 ± 55.7) in week five, then (849.3 ± 127.645.0) in three and (945.0 ± 72.6) in week one and the highest (1392.0 ± 194.1) in week two. In addition, five pollinator families (Crabronidae, Halictidae, Sphecidae, Tachinidae, and Vespidae) were prominent and captured every week.

Totaling the number of pollinators on sticky traps for each cowpea variety over the sampling weeks indicate no significant difference in the number of pollinators (*F*_23, 72_ = 0.85, *p* = 0.66) among the 24 cowpea varieties (Figure 3f). However, the number of pollinators was highest in Zipper Cream (219.5 ± 27.9), Whippoorwill (214.3 ± 19.1) and Mississippi Silver (205.5 ± 8.5) and lowest in Iron and Clay (106.5 ± 17.9) and Peking Black (143.5 ± 11.7) (Figure 3f).

#### 3.3.3. Evaluation of Pollinators from Direct Visual Counts

**Figure 4:**
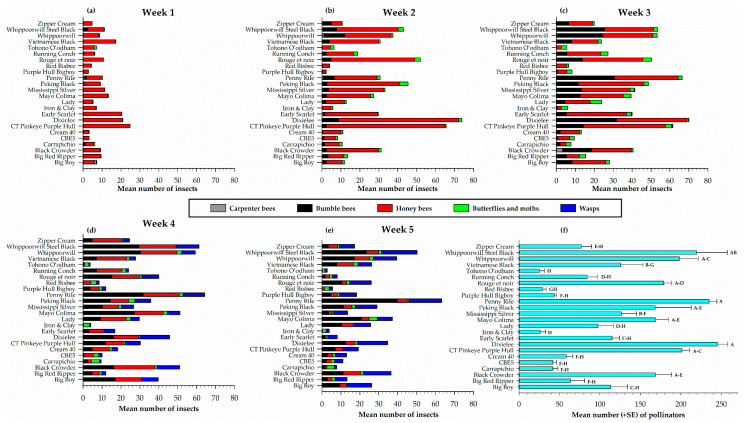
Weekly distribution of pollinator groups (**a**–**e**) and total number of pollinators (**f**) on different cowpea varieties through direct visual counts. In Figure 4f, mean numbers followed by the same letters are not significantly different (*p* > 0.05).

The weekly visual direct counts of the distribution and diversity of pollinators on the 24 cowpea varieties presented in Figure 4 indicates that three types of bees (honey bees, carpenter bees and bumble bees) were very prominent. Wasps, and butterflies and moths were also observed for a total of five different pollinator types (Figure 4). Over the entire sampling period, there were more honey bees observed, followed by bumble bees. All five pollinator types were recorded on all the 24 cowpea varieties; however, the distribution and diversity varied weekly. In week one, there were more honey bees than any other pollinator type on all the cowpea varieties, with the highest recorded on CT Pinkeye Purple Hull (24.8 ± 5.0), Dixielee (20.5 ± 10.0) and Early Scarlet (20.5 ± 7.4) (Figure 4a). In the second week, honey bees were still the major pollinator observed in the presence of a few bumble bees, butterflies and moths (Figure 4b). The distribution varied among the cowpea varieties with more observed on Dixielee (63.3 ± 4.2), CT Pinkeye purple Hull (62.8 ± 9.9), Rouge et noir (44.3 ± 11.6) and Whippoorwill Steel Black (32.5 ±14.4) (Figure 4b). By week three, there was a steady increase in the number of pollinators with more honey bees and bumble bees observed on each cowpea variety; however, Red Bisbee recorded no bumble bees and a few (≤ 3.3 ± 3.3) carpenter bees were observed on Black Crowder, Big Boy, Lady, Mississippi Silver, Penny Riley, Red Bisbee, Vietnamese Black Whippoorwill, and Zipper Cream (Figure 4c). The number of honey bees observed on each of the cowpea varieties had declined by week four; the presence of wasps was very noticeable on all the varieties the highest recorded on Dixielee (15.8 ± 3.3), Black Crowder (12.0 ± 3.3), Whippoorwill Steel Black (12.0 ± 4.4) and Penny Rile (11.5 ± 2.0) (Figure 4d). On the last sampling week, the number of honey bees had further declined and an increase in the number of wasps was observed. In contrast, the number of pollinators on each cowpea variety had decreased and Penny Riley (63.3 ± 6.8) Whippoorwill Steel Black (50.3 ± 10.3), and Whippoorwill (39.5 ± 5.9) still recorded the highest number of pollinators (Figure 4e). Overall, the weekly pollinator count was significantly different (*F*_4, 15_ = 9.80, *p* = 0.0004) among the five weekly samples.

A combined count of pollinators observed on each cowpea variety over the five-week sampling period, shows a significant difference (*F*_23, 72_ = 15.42, *p* < 0.0001) in the number of pollinators among the cowpea varieties (Figure 4f). Dixielee (245.3 ± 12.3), Penny Rile (235.0 ± 9.5) and Whippoorwill Steel Black (219.3 ± 38.0) (Figure 4f) had the highest counts.

**Figure 5:**
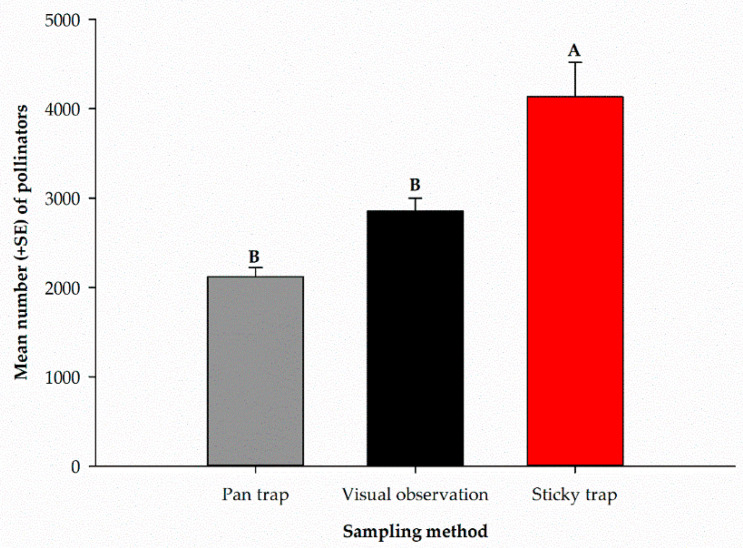
Mean number of all pollinators captured using different sampling methods over the entire sampling period. Mean numbers followed by the same letters are not significantly different (*p* > 0.05).

The mean number of pollinators captured by each sampling method over the entire sampling period is presented in Figure 5. There were significant differences (*F*_2, 9_ = 17.19, *p* = 0.0008) among the different sampling methods used. The highest number of pollinators was recorded in sticky traps (4132.3 ± 386.2), and the lowest number was captured in pan traps (2119.0 ± 104.7). Visual observation recorded (2853.8 ± 145.2) pollinators (Figure 5).

**Figure 6:**
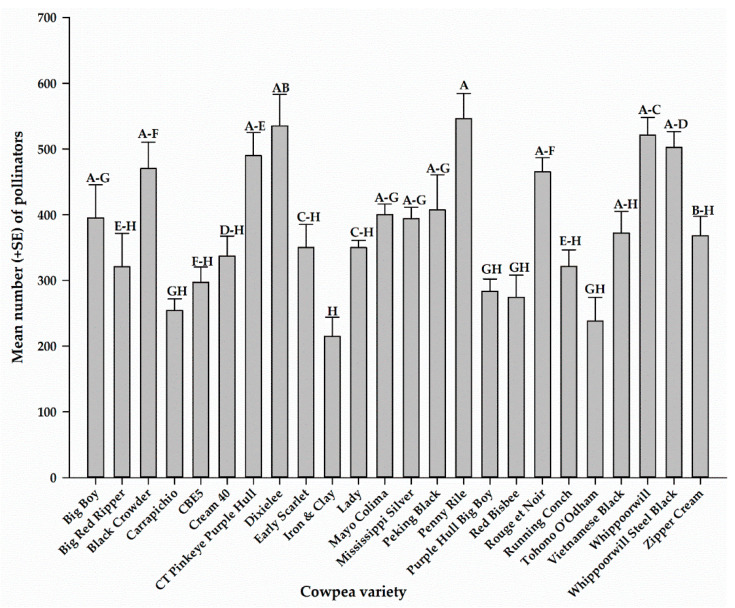
Mean number of all pollinators captured on each variety for the entire sampling period. Mean numbers followed by the same letters are not significantly different (*p* > 0.05).

Combining all pollinators from all sampling methods, the total number of pollinators captured on each cowpea variety for the entire sampling period is presented in Figure 6. There were significant differences (*F*_23, 72_ = 8.56, *p* < 0.0001) in the number of pollinators among the 24 cowpea varieties. The highest number of pollinators was recorded on Penny Rile (546.0 ± 38.6), and the lowest number was recorded on Iron and Clay (214.8 ± 29.2) (Figure 6).

### 3.4. Pollinator Abundance and Diversity Indices Associated with Cowpea Varieties and the Three Sampling Methods

A total of 16,529 insects representing 16 families were collected from sticky traps on all the 24 cowpea varieties over the entire sampling period revealed. The number of Tachinidae recorded on each cowpea variety averaged about 589 insects compared to an average range of 0.1 to 38 insects per variety for the remaining families. Therefore, Tachinidae was not included in the Diversity Index calculations. A total of 2389 insects representing 15 families was used. From visual observations, 11,415 insects, representing five pollinator types, were recorded. Of the 11 insect families identified from all pan traps, the number of Halictidae recorded on each cowpea variety averaged about 183 insects compared to an average range of 2–59 insects per cowpea variety for the other families. The family Halictidae was, therefore, not included in the Diversity Index calculations; a total of 4078 insects representing 10 families was used. The Diversity index (H’) and Evenness Indices (E) of insects captured in pan traps, sticky traps or counted visually on each of the cowpea varieties are shown on Table 4. When pan traps were used, insects were highly diverse and evenly distributed on Purple Hull Big boy (H′ = 1.94; E = 0.84) and less so on Tohono O’odham (H′ = 1.46; E = 0.63). For sticky traps, both indices were found to be highest on Penny Rile (H’ = 1.79; E = 0.66) and lowest on Tohono O’odham (H’ = 1.02; E = 0.38). Diversity indices ranged from 0.79 (Early Scarlet) to 1.25 (Big Red Ripper) in insects recorded through direct visual counts. In case of evenness, the highest value (0.82) was recorded on Purple Hull Big Boy and the lowest (0.37) on Running Conch. Overall, the lowest diversity was noticed when insect sampling was done through direct visual counts, and sampling with pan traps provided the highest Diversity Indices. These results show that the evenness of insect distribution as generally lowest in sticky traps where the values ranged from 0.38 to 0.66, compared to the other sampling methods. Tohono O’odham appears to be the cultivar with the lowest diversity, ranking lowest in two of the three sampling methods used.

### 3.5. Relationship between Number of Pollinators, Cowpea Flowers and Yield

**Figure 7:**
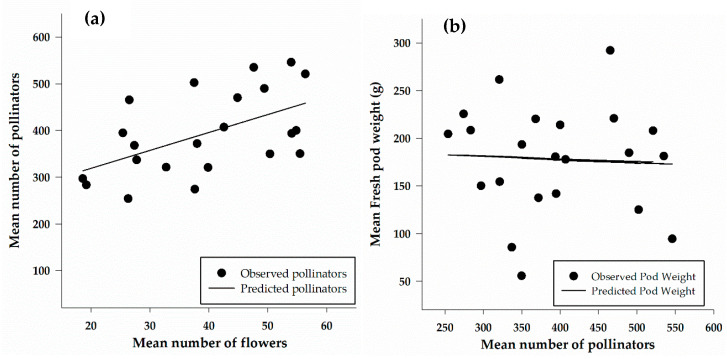
Linear regression showing the relationship between number of pollinators and number of flowers (**a**) (*y* = 242.09 + 3.8x; *n* = 22; *r*^2^ = 0.3; *p* = 0.01) and between fresh pod weight and number of pollinators (**b**) (*y* = 193.72 − 0.04x; *n* = 22; *r*^2^ = 0.004; *p* = 0.8).

A linear regression was calculated to show the relationship between the number of flowers and the number of pollinators captured in traps (Figure 7a). The number of pollinators increased linearly with an increase in the number of flowers. The relationship between the number of flowers and the number of pollinators caught in traps was moderate and significant (*F*_1, 20_ = 8.13, *p* = 0.01), with a correlation (*r*) of 0.54. The results of the regression indicated that approximately 30% of the variation in the number of pollinators is explained by the number of flowers. The relationship between yield (as pod weight) and the number of pollinators (Figure 7b) is weak (*r* = 0.06), non-significant (*F*_1, 20_ = 0.08, *p* = 0.8) relationship between yield and the number of pollinators.

## 4. Discussion

We have demonstrated in this study, through the use of three sampling methods including pan traps, sticky traps and direct visual counts, that some cowpea varieties could be used to increase and improve the abundance and diversity of pollinators. Each method provided a unique picture of pollinator diversity and abundance among the cowpeas. The most prominent pollinator in pan traps was Halictidae, while Tachinidae was the most abundant in sticky cards, and honey bees the most recorded from direct visual counts. The reason for this may likely be because honey bees are easily identifiable and large enough to be counted. Nonetheless, these findings confirm previous studies that reported that honey bees were the most abundant pollinators on cowpeas and other crops from direct visual counts [26,39,41,42,43,44]. The highest number of pollinators were recorded from sticky traps as a result of the high numbers of Tachinidae and this is similar to results from cornfields in Iowa, where more Tachinidae were captured by sticky cards compared to pan traps [37]. From several cowpea studies, tachinid fauna has been reported to attack cowpea pests such as the pod-sucking bug complex and cowpea curculio [45]. On the other hand, pan traps recorded more Halictidae similar to other studies that reported pan trap effectiveness at attracting Halictidae [36,37,41] compared to the other sampling methods. Sticky cards may not be good at capturing pollinators that are large in size such as honey bees, and pan traps may be an ineffective tool for capturing these bees when they are foraging.

Among the three colored traps used in our study, there were differences in pollinator abundance and diversity among cowpea varieties. White pan traps caught the highest number of pollinators on each of the 24 cowpea varieties (Figure 1b) and among the three colored traps (Figure 2b). The most abundant pollinator family in each of the pan traps among the cowpea varieties was the family Halictidae, with white pan traps recording the highest capture among the varieties (Table 3). Generally, pan traps were reported to be effective at catching halictid bees; however, after comparing white, yellow and blue pan traps, [36] reported the highest capture of pollinators in the family Halictidae in the white pan trap, similar to our findings, and [33] suggested white pan traps were the best color traps for sampling Halictidae. Contrary to these findings, [46] reported the highest capture of Halictidae in blue colored pan traps. In addition, according to [38], who studied the diversity of bees in a USA deciduous forest, yellow pan traps captured the most bees and blue was the least. This clearly suggests that the effectiveness of pan traps varies not only with color, but also in relation to habitat with respect to spatial and temporal factors. In order to capture a diverse sample of bees [47] recommended using a combination of blue, yellow, and white pan traps. Alternatively, for larger bees, our findings suggest the use of direct visual count to be effective.

Pollination of flowering plants represents a critical ecosystem service. Plant–pollinator interactions are usually mutual, with pollinators foraging for floral resources such as nectar and pollen that are used as food and in the process transfer pollen from anthers to stigmas thereby facilitating plant reproduction. About 75% of crops rely partially or completely on insects for pollination, accounting for ~$600 billion worth of annual global food production [48]. The decline in the abundance and diversity of pollinators has been reported to be related to several factors including loss of floral habitats [20] and this has subsequently resulted in a negative impact on crop yield [49]. Cowpea is highly nectariferous and attractive to bees and other pollinators [26]. It is a self-pollinating crop [50], nonetheless, cross pollination occurs at a minimal extent (1 and 10%) depending on the pollinator type, the environment, and variety [39,51]. Insects may, therefore, be visiting cowpeas for nectar and/or pollen and not necessarily to pollinate the crop.

Although many insect species are known to provide pollination services, honey bees and other bees are often assumed to be the main providers of these services in agricultural ecosystems [52]. For example, several crops including buckwheat [53] almond, onion, carrot, and tomatoes [54] are pollinated typically and commercially by Apidae. To achieve this, many growers in the USA rent honey bee colonies to pollinate their crops. Approximately 2.5 million commercially managed honey bee colonies are used for crop pollination yearly in the USA [55]. We recorded five pollinator types (honey bees, carpenter bees, bumble bees, butterflies and moths, and wasps) from direct visual counts, with honey bee the most prominent flower visitor. These findings are similar to insects observed on some local cowpea varieties [26,39,42,43,44]. The abundance of Apidae varied among cowpeas varieties with CT Pinkeye Purple Hull, Whippoorwill Steel Black, Whippoorwill, Dixielee and Penny Rile recording the highest and Carrapichio, CBE5, Iron and Clay, Purple Hull Big Boy, Red Bisbee, Tohono O’Odham the least. The foraging activities of honey bees are greatly influenced by several factors including the availability of nectar and pollen [26]. A study by [43], showed that the amount of pollen produced varied in size and quantity among three cowpea varieties. It could be possible that these factors contributed to the distribution of pollinators among the cowpea varieties in our study. Growers can be encouraged to grow pollinator dependent crops in a cropping system that would integrate cowpea varieties reported in our study to be highly attractive to honey bees. Such a system would be profitable to the grower and, at the same time, improve honey bee health and abundance.

The global reliance on honey bees as the single most effective pollinator species is a risky strategy, especially given the current global trends showing a decline in honey bees resulting from poor nutrition, ectoparasitic mite *Varroa destructor* and a number of other pests and diseases [56]. However, honey bees are not the only insects that pollinate crops and knowledge of other bee species is increasing. The family Halictidae is one of the most diverse among native bees and is also one of the most important pollinators for crops such as stone fruits (peach, almond, plum, cherry and apricot), pome fruits (apple, pear, and quince), sunflowers, clover, alfalfa, and some wildflowers. From our results, the abundance and distribution of Halictid bees varied among cowpea varieties with Black Crowder, CT Pinkeye Purple Hull, Dixielee, Early Scarlet, Penny Rile, Rouge et Noir, Whippoorwill and Whippoorwill Steel Black recording the highest number. We, therefore, have specific cowpea varieties that can be intercropped with a specific pollinator dependent crop to enhance crop yield. According to [57], pollinators in the family Halictidae were important in eggplant production in Brazil and in the cultivation of lettuce flower for seed [58]. Halictidae have also been reported to be important for pollination of melon [59], and in *Prescottia densiflora*, a flowering plant from the orchid family [60]. Some studies involving intercropping similar pollinator-dependent crops with cowpea have reported different results. For example, an increase in crop yield was reported from intercropping tomato and cowpea [31,61] and okra and cowpea [31]. Meanwhile, in another study, intercropping tomato and okra with cowpea caused between 45 and 55% loss in marketable fruit yields in tomato and okra, respectively, and cowpea grain yield was reduced up to 55% by intercropping [62]. In such intercropping, the yield increases were not only due to improved nitrogen nutrition of the cowpea component and reduced pest damage but also to other unknown factors which could include the presence of pollinators. The impact of pollinator on yield could be attributed to the use of cowpea varieties that attract pollinators not beneficial to the crop production system or cowpeas that do not attract pollinators. From our results, specific cowpea varieties can be intercropped with specific pollinator dependent crops to enhance yield.

Flower color is one of the most important visual traits exploited by pollinating insects [63]. The display of colorful flowers triggers behavioral responses and serve as sensory signals that attract flower visitors by signaling the quality and quantity of floral rewards [64,65]. Some studies have demonstrated that pollinators rely strongly on color to make their foraging decisions [66,67]. The twenty-four varieties used in this study can be grouped into three colors as either yellow, purple/violet, or white. Early Scarlet is the only cowpea variety in our study that produce yellow flowers and had the shortest number days to flower compared to the other varieties (Table 1). However, it was not among the most attractive to pollinators. This could be due to the phenology of Early Scarlet cowpea and the time of pollinator appearance. Early scarlet produced more flowers forty-two days after planting and within two weeks pods developed (Table 1). From visual observation (Figure 4), there were more honey bees reported on Early Scarlet within the first thee sampling weeks, a time frame that corresponds to the presence of profuse flowers. Running Conch, Big Boy, Purple Hull Big Boy, Zipper cream, Lady and Cream 40 were varieties that produce white flowers but their attractiveness to pollinators varied significantly (Figure 6). The other cowpea varieties produce purple/violet color flowers; a color reported to be highly attractive to bees [68]. Penny Rile, Dixielee, CT Pinkeye purple Hull, Whippoorwill Steel Black and Whippoorwill all have purple/violet-colored flowers and recorded the highest number of pollinators. In contrast, other varieties such as CBE5, Carrapichio and Red Bisbee also with purple/violet color recorded fewer pollinators. Clearly, flower color may not be the only clue used by all pollinators but an important component of the retinue of stimuli that determine the choice of a plant for pollination. Nectar, pollen, and odor may play combined or overriding rewards for flowers preference or visitation.

Other workers have suggested that flower number (resource concentration) may also be critical in attraction [69,70,71,72,73]. These studies have shown that an increase in the number of inflorescences resulted in an increase in pollinator visits and [71] attributed this to increasing attractiveness. Several studies in urban ecology areas reported floral abundance to have a positive influence on the pollinators and significantly influenced bees [74,75,76,77,78,79], hoverflies [77,78], and butterflies [76,80]. Although our results show there was a significant positive relationship between the number of pollinators and the number of flowers, we found that varieties such as Penny Rile, Dixielee, CT Pinkeye purple Hull, Whippoorwill Steel Black and Whippoorwill that recorded high numbers of pollinators were not among those that produced the most flowers. Additionally, Early Scarlet, Mayo Colima, and Lady were among varieties with the highest number of flowers but not the most attractive to pollinators. Early Scarlet flowered early and converted into pods early. This may have had an effect on the total number of pollinators recorded on this variety. Tohono O’Odham and Iron and Clay produced no flowers but still attracted some pollinators albeit very low numbers (Figure 6). From our results, the presence of flowers does not necessarily attract pollinators; other factors such as the quantity and/or quality of nectar, and other floral-related traits, may also contribute to the attractiveness of insect pollinators to flowers [39,81,82].

## 5. Conclusions

Pan traps and sticky traps remain effective sampling tools for estimating the diversity of insects such as pollinators. However, direct visual counts are a quicker sampling method when targeting specific insect groups that are easily assessed. A combination of these sampling methods may provide detailed information on insect diversity in the community and thus particularly useful in pollination studies. Cowpea is a crop that has traditionally been grown as an intercrop, mainly as a source of protein. In some cases, where cowpea has been used as an intercrop, it serves to manage insect pests and diseases as well as improve soil fertility and control weeds, culminating in increased crop yield [83]. Its use for pollination-dependent crops is recent. Adding cowpea to cropping systems provides increased benefits when the right types of pollinators are attracted to the pollinator-dependent crop. Our results indicate that pollinator abundance and diversity vary among the different cowpea varieties thus supporting the use of specific cowpea variety with a specific pollinator-dependent crop to enhance pollination. Therefore, to get maximum benefits to the intercrop, it is important to select cowpea varieties that would produce optimal attraction for respective crop combinations. For instance, our study shows that honey bees were one of the most abundant pollinators recorded on some cowpeas, thus crops that are typically pollinated by honey bees should be intercropped with cowpea varieties such as Penny Rile and Purple Hull Big Boy that recorded the highest number of honey bees, to help increase the yield of these crops.

## Figures and Tables

**Table 1:** Days to first flower and first pod formation, number of flowers, and color of the 24 cowpea varieties studied. Means numbers followed by the same letters are not significantly different (*p* > 0.05). The number of days to first pod was not different among the varieties.

Variety	Mean # (±SE) of Days to First Flower	Mean # (±SE) of Flowers/Plant	Mean # (±SE) of Days to First Pod	Flower Color
Big Boy	54.5 ± 1.5 a	25.4 ± 1.5 fg	62.8 ± 2.5	White
Big Red Ripper	51.0 ± 2.2 ab	39.9 ± 4.0 a–f	62.8 ± 2.5	Purple
Black Crowder	52.8 ± 1.4 a	44.9 ± 4.5 a–e	61.0 ± 2.1	Purple
Carrapichio	50.3 ± 1.7 ab	26.3 ± 2.4 fg	64.5 ± 2.5	Purple
CBE5	55.5 ± 3.7 a	18.6 ± 1.5 g	64.5 ± 2.5	Purple
Cream 40	54.0 ± 4.0 a	27.8 ± 2.7 e–g	64.5 ± 2.5	White
Ct Pinkeye Purple Hull	45.5 ± 1.3 ab	49.4 ± 3.5 a–d	57.5 ± 2.5	White
Dixielee	49.3 ± 1.4 ab	47.6 ± 2.9 a–d	62.8 ± 1.7	Purple
Early Scarlet	42.0 ± 1.2 b	55.5 ± 3.9 ab	57.5 ± 2.5	Yellow
Iron & Clay	-	-	-	-
Lady	51.3 ± 1.8 ab	50.4 ± 4.4 a–d	61.0 ± 2.1	White
Mayo Colima	48.3 ± 0.8 ab	54.8 ± 4.0 a–c	61.0 ± 2.1	Purple
Mississippi Silver	50.3 ± 2.8 ab	54.1 ± 4.5 a–c	61.0 ± 2.1	Purple
Peking Black	49.3 ± 1.4 ab	42.6 ± 4.6 a–f	61.0 ± 2.1	Purple
Penny Rile	51.3 ± 1.0 ab	54.0 ± 5.4 a–c	61.0 ± 2.1	Purple
Purple Hull Big Boy	55.5 ± 2.1 a	19.2 ± 1.3 g	64.5 ± 2.5	White
Red Bisbee	54.8 ± 0.8 a	37.6 ± 4.3 b–f	64.5 ± 2.5	Purple
Rouge et Noir	48.3 ± 0.8 ab	26.5 ± 2.2 fg	61.0 ± 2.1	Purple
Running Conch	50.3 ± 1.7 ab	32.8 ± 3.6 d–g	61.0 ± 2.1	White
Tohono O’odham	-	-	-	-
Vietnamese Black	47.3 ± 1.3 ab	38.0 ± 4.5 b–f	61.0 ± 2.1	Purple
Whippoorwill	49.3 ± 1.4 ab	56.4 ± 4.2 a	59.3 ± 0.8	Purple
**Whippoorwill Steel Black**	53.0 ± 2.9 a	37.5 ± 3.2 c–f	61.0 ± 2.1	Purple
**Zipper Cream**	52.8 ± 2.4 a	27.4 ± 2.7 e–g	62.8 ± 2.5	White

**Table 2:** Mixed model analysis for sampling method, sampling week, and cowpea varieties on abundance of pollinators.

Source	DF	L-R χ2	Prob > χ2
Sampling week	4	167.35628	<0.0001
Sampling methods	4	645.27582	<0.0001
Sampling week × Sampling methods	16	87.586056	<0.0001
Variety	23	84.183526	<0.0001
Sampling week × Variety	92	35.250846	1.000
Sampling method × Variety	92	140.18891	0.0009
Sampling week × Sampling method × Variety	368	95.557676	1.000

**Table 3:** Diversity and abundance (mean ± SE) of pollinators per pan traps on cowpea varieties over a five-week sampling period. Means numbers followed by the same letters within rows are not significantly different (*p* > 0.05).

	Mean number per trap ± SE											
	White				Yellow				Blue			
Variety	Apidae	Crabronidae	Halictidae	Tachnidae	Apidae	Crabronidae	Halictidae	Tachnidae	Apidae	Crabronidae	**Halictidae**	**Tachnidae**
Big Boy	1.3 ± 0.5 b	15.0 ± 6.0	32.5 ± 6.1	5.3 ± 1.7	0.5 ± 0.5	2.8 ± 0.9 b	9.5 ± 2.0 ab	3.0 ± 1.8	1.0± 0.7	0.3 ± 0.3	7.0 ± 2.1	1.3 ± 0.5
Big Red Ripper	1.0 ± 1.0 b	8.8 ± 0.3	32.3 ± 10.4	6.3 ± 2.2	0.0 ± 0.0	1.5 ± 1.0 b	6.0 ± 2.1 b	3.8 ± 0.9	2.3 ± 1.3	0.5 ± 0.5	8.0 ± 1.1	1.5 ± 0.9
Black Crowder	3.3 ± 1.3 b	14.3 ± 5.3	35.8 ± 8.3	4.8 ± 2.5	2.5 ± 2.2	5.0 ± 1.2 a	17.3 ± 3.2 a	6.5 ± 2.7	7.3 ± 2.9	1.0 ± 0.7	6.8 ± 2.1	1.3 ± 0.5
Carrapichio	0.3 ± 0.3 b	5.8 ± 2.7	22.8 ± 5.5	3.0 ± 1.1	0.5 ± 0.3	1.5 ± 0.6 b	8.3 ± 2.3 b	1.8 ± 1.8	2.8 ± 0.8	1.0 ± 1.0	5.8 ± 1.8	0.3 ± 0.3
CBE5	0.5 ± 0.3 b	9.3 ± 2.1	34.0 ± 9.7	10.5 ± 2.0	0.3 ± 0.3	1.3 ± 0.6 b	9.8 ± 1.4	3.8 ± 1.7	1.3 ± 0.5	0.3 ± 0.3	5.8 ± 2.8	1.0 ± 0.7
Cream 40	0.8 ± 0.3 b	9.3 ± 3.6	29.5 ± 7.3	3.5 ± 2.3	0.5 ± 0.3	3.8 ± 1.8 b	12.8 ± 2.4	3.8 ± 2.2	2.0 ± 0.4	0.3 ± 0.3	4.0 ± 1.4	0.0 ± 0.0
CT Pinkeye Purple Hull	1.3 ± 0.8 b	25.3 ± 17.0	35.0 ± 1.8	5.5 ± 2.4	0.3 ± 0.3	2.3 ± 0.5 b	9.0 ± 1.9 b	1.5 ± 0.6	4.8 ± 1.8	1.3 ± 0.8	10.5 ± 1.3	1.5 ± 1.5
Dixielee	6.5 ± 4.6 a	14.0 ± 2.9	35.0 ± 6.8	4.0 ± 2.7	2.0 ± 0.9	5.5 ± 1.8 b	12.8 ± 1.3	7.5 ± 2.7	2.8 ± 1.0	0.5 ± 0.3	5.8 ± 3.4	0.5 ± 0.3
Early Scarlet	1.0 ± 0.7 b	6.0 ± 1.8	25.0 ± 5.0	4.0 ± 1.2	1.3 ± 0.3	1.8 ± 0.9 b	14.3 ± 1.5 a	1.5 ± 0.9	1.8 ± 0.3	0.3 ± 0.3	6.5 ± 2.1	1.0 ± 1.0
Iron and Clay	0.5 ± 0.5 b	11.3 ± 3.2	26.3 ± 4.6	7.8 ± 3.3	0.5 ± 0.3	2.5 ± 1.5 b	9.8 ± 2.1 b	2.8 ± 2.8	1.3 ± 0.8	0.3 ± 0.3	6.8 ± 1.1	0.3 ± 0.3
Lady	2.3 ± 1.9 b	7.8 ± 3.1	26.0 ± 7.6	2.5 ± 0.5	0.0 ± 0.0	2.8 ± 1.5 b	13.8 ± 4.4 a	2.8 ± 2.4	1.3 ± 0.6	0.3 ± 0.3	6.0 ± 1.4	0.5 ± 0.3
Mayo Colima	2.3 ± 0.8 b	13.3 ± 5.3	31.8 ± 9.1	7.5 ± 2.2	0.3 ± 0.3	1.5 ± 0.6 b	6.8 ± 1.3 b	4.0 ± 2.7	1.8 ± 0.6	0.0 ± 0.0	4.3 ± 1.3	0.5 ± 0.3
Mississippi Silver	1.0 ± 0.7 b	1.5 ± 0.9	24.5 ± 2.1	2.5 ± 0.9	0.5 ± 0.3	2.5 ± 1.7 b	9.8 ± 1.4 ab	3.8 ± 2.8	2.5 ± 1.2	1.5 ± 1.2	5.8 ± 0.9	0.0 ± 0.0
Peking Black	2.5 ± 1.2 b	20.5 ± 6.1	38.0 ± 1.5	6.5 ± 1.8	0.5 ± 0.5	0.8 ± 0.3 b	8.0 ± 1.8 b	1.0 ± 0.6	3.5 ± 1.0	1.0 ± 0.7	3.3 ± 1.0	0.5 ± 0.5
Penny Rile	10.0 ± 4.1 a	18.3 ± 5.0	34.5 ± 7.7	9.0 ± 0.8	3.0 ± 0.9	7.3 ± 2.1 a	14.0 ± 0.8 a	4.8 ± 2.1	3.3 ± 1.7	0.8 ± 0.5	7.5 ± 1.7	0.3 ± 0.3
Purple Hull Big boy	1.3 ± 0.8 b	5.0 ± 1.3	25.0 ± 3.3	3.3 ± 0.8	3.8 ± 2.8	2.3 ± 1.1 b	10.3 ± 2.6 ab	2.3 ± 1.4	1.8 ± 0.5	0.5 ± 0.3	6.0 ± 1.7	0.8 ± 0.3
Red Bisbee	1.5 ± 0.5 b	15.8 ± 4.6	28.0 ± 4.8	6.0 ± 1.1	0.5 ± 0.3	0.3 ± 0.3 b	8.8 ± 1.0 b	2.5 ± 1.2	0.8 ± 0.5	0.0 ± 0.0	3.8 ± 0.5	0.0 ± 0.0
Rouge et noir	4.8 ± 3.8 b	14.3 ± 3.9	36.5 ± 17.3	5.5 ± 3.6	1.5 ± 1.2	4.3 ± 2.5 b	17.5 ± 4.9 a	2.8 ± 1.2	3.5 ± 2.6	1.0 ± 1.0	6.0 ± 1.1	1.5 ± 0.3
Running Conch	0.5 ± 0.3 b	3.5 ± 1.0	20.3 ± 5.0	1.5 ± 0.6	0.5 ± 0.3	1.8 ± 1.2 b	6.5 ± 1.9 b	2.0 ± 1.7	1.5 ± 0.3	0.5 ± 0.3	4.5 ± 1.7	0.0 ± 0.0
Tohono O’odham	0.3 ± 0.3 b	6.3 ± 1.4	20.0 ± 4.4	4.5 ± 1.0	0.0 ± 0.0	0.5 ± 0.5 b	5.0 ± 1.8 b	0.8 ± 0.3	0.5 ± 0.3	0.3 ± 0.3	4.0 ± 0.9	0.0 ± 0.0
Vietnamese Black	1.8 ± 0.5 b	14.8 ± 6.0	22.0 ± 4.8	6.0 ± 2.7	1.5 ± 1.2	1.3 ± 0.6 b	8.3 ± 1.4 b	3.0 ± 0.9	2.5 ± 1.6	1.0 ± 0.7	5.0 ± 2.3	0.3 ± 0.3
Whippoorwill	4.8 ± 2.8 b	15.5 ± 6.9	34.5 ± 8.2	10.0 ± 3.7	0.8 ± 0.8	2.0 ± 1.4 b	6.8 ± 3.3 b	8.0 ± 4.6	5.0 ± 2.7	0.5 ± 0.3	7.0 ± 2.1	1.0 ± 0.6
Whippoorwill Steel Black	8.5 ± 3.3 a	17.3 ± 8.2	39.8 ± 9.6	10.3 ± 3.8	3.3 ± 2.6	3.3 ± 1.4 b	11.0 ± 1.4 ab	7.3 ± 3.8	3.3 ± 2.3	0.8 ± 0.3	9.0 ± 2.0	2.8 ± 1.4
Zipper Cream	0.5 ± 0.3 b	6.5 ± 2.6	20.0 ± 2.9	3.0 ± 1.2	7.0 ± 6.7	3.0 ± 1.6	9.5 ± 2.6 ab	1.8 ± 0.8	2.3 ± 0.9	1.0 ± 0.4	7.0 ± 2.7	1.0 ± 0.4
***F value***	*1.97*	*1.56*	*0.83*	*1.5*	*0.91*	*1.84*	*1.98*	*1.13*	*1.27*	*0.76*	*0.85*	*1.35*
***P value***	*0.02*	*0.08*	*0.69*	*0.95*	*0.58*	*0.03*	*0.02*	*0.34*	*0.22*	*0.77*	*0.66*	*0.17*

**Table 4:** Diversity index (H′) and Evenness (E) of insect families in pan traps, sticky traps, and from direct visual counts recorded on cowpea varieties.

Variety	Pan Trap		Sticky Trap		Direct Visual Counts	
	H′	E	H′	E	H′	E
Big Boy	1.71	0.74	1.50	0.55	1.22	0.76
Big Red Ripper	1.87	0.81	1.46	0.54	1.25	0.78
Black Crowder	1.86	0.81	1.56	0.57	1.16	0.72
Carrapichio	1.89	0.82	1.42	0.53	1.20	0.74
CBE5	1.78	0.77	1.38	0.51	1.17	0.72
Cream 40	1.56	0.68	1.41	0.52	1.11	0.69
CT Pinkeye Purple Hull	1.55	0.67	1.58	0.58	0.84	0.52
Dixielee	1.76	0.76	1.66	0.61	1.03	0.64
Early Scarlet	1.86	0.81	1.24	0.46	0.79	0.49
Iron and Clay	1.75	0.76	1.44	0.53	1.00	0.62
Lady	1.85	0.80	1.34	0.50	1.24	0.77
Mayo Colima	1.73	0.75	1.58	0.58	1.15	0.72
Mississippi Silver	1.85	0.80	1.24	0.46	0.87	0.54
Peking Black	1.59	0.69	1.45	0.53	1.12	0.70
Penny Rile	1.81	0.79	1.79	0.66	1.13	0.71
Purple Hull Big Boy	1.94	0.84	1.57	0.58	1.31	0.82
Red Bisbee	1.62	0.70	1.59	0.59	1.00	0.62
Rouge et noir	1.87	0.81	1.57	0.58	1.08	0.40
Running Conch	1.70	0.74	1.56	0.58	1.00	0.37
Tohono O’odham	1.46	0.63	1.02	0.38	1.09	0.68
Vietnamese Black	1.84	0.80	1.37	0.51	0.99	0.62
Whippoorwill	1.75	0.76	1.64	0.60	1.13	0.70
Whippoorwill Steel Black	1.78	0.77	1.61	0.59	1.13	0.70
Zipper Cream	1.79	0.78	1.41	0.52	1.10	0.69

## Data Availability

Data sharing not applicable.
